# “Someone could have told me”: a qualitative study on experiences with hysterectomy decision-making and regret in Mysore, India

**DOI:** 10.3389/fgwh.2026.1870806

**Published:** 2026-08-10

**Authors:** Sasha Herbst de Cortina, Raghavi Karanam, Rashmi Pramathesh, Fazila Begum, Poornima Jaykrishna, Vijaya Srinivas, Roopa Hariprasad, Purnima Madhivanan, Prajakta Adsul, Devanshi Somaiya

**Affiliations:** 1 Division of Infectious Diseases, Department of Medicine, University of California Los Angeles, Los Angeles, CA, United States; 2 Public Health Research Institute of India, Mysore, Karnataka, India; 3 Centre for Evidence for Guidelines, Department of Health Research, Indian Council of Medical Research, Ministry of Health and Family Welfare, New Delhi, India; 4 Health Promotion Sciences Department, Mel & Enid College of Public Health, University of Arizona, Tucson, AZ, United States; 5 Division of Epidemiology, Biostatistics, and Preventive Medicine, Department of Medicine, University of New Mexico, Albuquerque, NM, United States; 6 Cancer Control and Population Sciences Research Program, Comprehensive Cancer Center, University of New Mexico, Albuquerque, NM, United States

**Keywords:** decisional regret, decisional support, decision-making, hysterectomy, India, qualitative research, women's health

## Abstract

**Introduction:**

Hysterectomy is a common surgery among women in India and has been normalized as a treatment for a range of conditions including those with alternative treatment options. There are concerns regarding its overuse, appropriateness, and potential contribution to poor health outcomes, especially among younger women. As with all surgical decision-making, it can be a challenge to balance patient autonomy with physician expertise in decision-making.

**Methods:**

We performed in-depth interviews with 20 women who had undergone hysterectomy prior to age 35 in semi-urban areas of Mysore District, Karnataka, India to gain insights around their decision-making experiences. Transcripts were evaluated using thematic analysis, and we used the Ottawa Decision Support Framework to frame our findings around three key domains: decision impact, status and gaps in decisional support, and ongoing decisional needs.

**Results:**

Participants described positive outcomes when personal values were aligned with decision-making, and regret when they were not aligned. They often felt under-supported in decision-making. Key resources in decision-making were from community members and medical personnel. Finally, participants identified a gap in formal support in place for women considering hysterectomy and highlighted the need for better counseling by medical providers.

**Discussion:**

Recommendations for improving decisional support and minimizing decisional regret include expanding education by community health workers regarding gynecologic health, personalized and accurate discussion of health conditions and personal values by physicians, increased government oversight of hysterectomy, and expanded access to routine healthcare to increase treatment options.

## Introduction

Hysterectomy, a surgery to remove the uterus and often other female reproductive organs, is a commonly performed surgery in India. According to the 2019–2021 countrywide Fifth National Family Health Survey (NFHS-5) conducted in India, 3.3% of Indian women currently aged 15–49 have had hysterectomies, although the prevalence varies significantly by region (1.2%–4.2%) and age group (0.2%–9.7%) (1). This is likely an underestimate, as data from other sources such as the Longitudinal Aging Study in India performed in 2017–2018, found a prevalence as high as 11.4% among women over age 45, or younger women with spouses over age 45 (2). While there are limited data on global prevalence of hysterectomy, the data from India are in line with data from other countries which report an approximately 10% prevalence of hysterectomy (3–5). While prevalence of a once-in-a-lifetime surgery naturally increases with increasing age of the cohort studied, notably in other countries, there is a significant trend towards higher rates of hysterectomy in women of older age (6–8). In contrast, India's NFHS-4 (performed in 2015–2016) found that among women reporting a hysterectomy, 62% were age 35 or younger when they underwent the surgery (9). There is a longstanding concern regarding overuse of this surgery, particularly for young women (10). Across India, hysterectomy is associated with potentially vulnerable demographic groups including women with lower education level, rural vs. urban residence, and lower caste (1, 2, 9, 11–14). Hysterectomy prevalence is notably higher in the southern region of India compared to other parts of the country (1). This regional disparity raises important questions about the underlying socio-cultural, economic, and health system factors influencing hysterectomy practices in different parts of the country.

Hysterectomy can be performed for life threatening indications such as uterine cancers, acute bleeding, and post-partum complications, as well as non-life-threatening reasons such as chronic pain or bleeding from uterine fibroids. In India, non-life-threatening indications predominate, with reasons commonly reported including menstrual bleeding, painful menses, fibroids, prolapsed uterus, abdominal pain, and abnormal vaginal discharge (2, 13, 15–21). The focus on hysterectomy in India for benign conditions among young women has prompted investigation into why women seek the surgery. In surveys of Indian women who have undergone a hysterectomy, factors include belief of hysterectomy as the primary treatment for gynecologic conditions, lack of awareness of other treatments, perception of the uterus as a useless organ after childbearing is complete, and fear of cancer developing if the uterus remains (17, 22, 23). In some areas, women specifically seek hysterectomy to avoid limitations placed on women during menstruation due to the idea of menstruation as a time of impurity in Hinduism, the predominant religion in India (15, 17, 24). In general, the high prevalence of hysterectomy may have created a societal norm encouraging women to seek hysterectomy as a definitive solution for gynecologic issues which affect their ability to work and quality of life (25).

Despite its normalization, hysterectomy is not appropriate for every patient or clinical scenario. Some of the common indications, for example abdominal pain or abnormal vaginal discharge, may not be related to the uterus, and would not necessarily be addressed with hysterectomy. For uterine fibroids and painful menstruation, hysterectomy is typically last-line, reserved for cases where symptoms significantly affect quality of life despite other therapies, and only in select patients for whom a major surgery is appropriate (26, 27). Although generally well-tolerated, hysterectomy is not necessarily harmless. It is associated with surgical complications including bleeding, damage to nearby organs, infections, adhesions, chronic abdominal pain, and incisional hernias (28). Hysterectomy is commonly performed concurrently with oophorectomy (removal of the ovaries). While there has not been widespread data collection on rates of oophorectomy with hysterectomy in India, smaller studies report up to 59% of hysterectomies also involving bilateral oophorectomy (29). This is particularly concerning due to implications for premature menopause and related health issues with oophorectomy. In the long term, especially for younger and pre-menopausal women, hysterectomy with or without oophorectomy has been linked to increased risk for neurologic disease including stroke and dementia, cardiovascular disease such as hypertension, diabetes, obesity, osteoporosis, premature menopause, sexual dysfunction, and mood dysfunction (1, 2, 30–38).

Worryingly, Indian women who have undergone a hysterectomy often report limited knowledge of the surgery or the exact body parts that were removed during their surgery (16). Women also report feeling rushed into the surgery without appropriately understanding the risks, benefits, and alternative options (10, 23, 39–41). Prior literature has highlighted the paternalistic and possibly predatory or coercive nature of providers recommending hysterectomy, often with pressure for urgent surgery (13, 22, 40). As hysterectomy is often covered by public Indian insurance plans, it is potentially seen as an easy source of revenue for providers (42).

The uncertainty regarding appropriateness of the surgery as well as women's limited knowledge about the surgery indicate a major gap in the current literature around the decision-making process that occurs prior to hysterectomy. As with many clinical conditions that require surgical decision-making, it can be a challenge for physicians to balance patient autonomy with physician expertise. For patients with low health literacy and limited financial resources, it is crucial to uphold principles of informed consent while not allowing this to limit access to necessary medical care. Better understanding of decision-making for hysterectomy in India is necessary for optimizing the use of this surgery and requires diverse input from those involved in the process. Given the complexity of major surgical decisions, deeper insight into decision-making is needed beyond quantitative data. Although large-scale surveys have shed light on prevalence and demographic patterns (1, 2, 9), they fall short of capturing the nuanced experiences, perceptions, and power dynamics involved in the decision-making process.

We therefore aimed to utilize qualitative inquiry to gain insights around decision-making among women who have undergone hysterectomy, using in-depth, semi-structured interviews.

## Materials and methods

### Setting and recruitment

This study was conducted in the Mysore District of the South Indian state of Karnataka. Early hysterectomy had been anecdotally suggested by members of the community as potentially an important component of women's health in this area. According to NFHS-4 (11), 3.37% of women in Karnataka, and approximately 4%–6% of women in Mysore District have had hysterectomies. In the southern region of India, hysterectomy prevalence was higher among rural women (6.9%), and less educated women (11.1% for those with no education, and 8.2% for those who attended only primary school). The median age at hysterectomy for women in Karnataka was 32 years old.

The Public Health Research Institute of India (PHRII) and University of California (UC) Berkeley Institutional Review Boards reviewed and approved the study prior to recruitment (PHRII protocol 2019-10-12-55 dated 12 October 2019, UC Berkeley protocol 2019-10-12599 dated 20 January 2020).

Participants were recruited from rural and semi-urban communities of Mysore District. PHRII has over two decades of experience working in these communities, and the team of community outreach workers, including authors RP and FB, have ongoing rapport and relationships with these populations. The authors of this study are all female and include Indian women born and raised in this community, women from other areas of India, women who are from India but now live full time in the United States, and one woman from the United States. All authors have written positionality statements which are available in the supplement. RP and FB reached out to individual women who had participated in or collaborated on prior PHRII projects to determine their eligibility. Once participants were identified, we used a snowball sampling approach by asking participants to identify additional eligible women in their communities. There were no refusals to participate documented. All potential participants were contacted in person in their villages.

Women were included in the study if they were between ages 18–50 and self-reported to have undergone hysterectomy at the age of 35 or younger. Women were excluded if they had a known gynecological cancer which necessitated a hysterectomy. All participants lived in the outlying villages of the Mysore District and provided written informed consent in *Kannada* to participate in the study. Interview themes were continuously assessed. After the initial two interviews were performed, the ice breaker question was changed due to difficulty capturing the concept in translation from English to *Kannada*. The remainder of the questions were found to be appropriate and used for all other interviews. Data saturation, where authors noted the same themes arising in interviews without new concepts, ideas, or themes emerging, was reached after 18 interviews, and recruitment was stopped. Due to the time required of transcription and translation, two additional interviews had already been performed for a total of 20 included in the analysis. This sample size lies within the general consensus range appropriate for qualitative work, especially given our relatively homogeneous study population and narrow focus on experience with hysterectomy (43, 44).

### Data collection and analysis

We used a qualitative approach because there was limited prior information about this subject in this context and in order to gain more nuanced, detailed insight into women's decision-making experiences. The COREQ checklist for qualitative research is available in the supplement (45). Because reproductive and sexual health can be sensitive topics, we interviewed women individually rather than in focus groups. We designed the interview guide (available in the supplement) to address two broad domains: the pre-hysterectomy decision-making process, and post-hysterectomy effects on health and lifestyle. The interview included questions about participants' general understanding of hysterectomy; reasons they underwent the surgery; how they came to the decision and who helped them; any changes in their physical, mental, and sexual health since the surgery; in what ways lack of menstruation and inability to get pregnant had affected their lives; how they felt about their decision now; and what advice they would give to other women and doctors regarding hysterectomy. Participants also completed a demographic questionnaire including when and where they underwent the hysterectomy. Each completed one interview which lasted approximately an hour.

Interviews took place in participants' homes. FB conducted interviews in the local language of *Kannada* with RP taking field notes. Both FB and RP are female community counselors at PHRII who have conducted prior in-depth interviews for qualitative studies. SH, also a woman, sat in for some interviews but did not speak *Kannada* and did not participate in the conversations. Interviews were audio-recorded, transcribed verbatim in *Kannada* by other members of the PHRII team, and translated into English using a professional translation service. FB and RP checked the English transcripts for accuracy without back translation or returning them to participants for review.

Transcripts were analyzed in Dedoose, a cloud application for managing, analyzing, and presenting qualitative and mixed method research data (version 9, 2021, Los Angeles, California, USA). We used Thematic Analysis described by Braun and Clarke, which is an iterative process where researchers get familiar with the data, generate codes, generate themes based on relationship within the codes, review themes, define and name them and finally present the themes using exemplar quotes (46). Authors SH and PA independently reviewed all transcripts and generated code lists inductively. They met routinely and utilized consensus coding in which they discussed transcripts and compared codes to create a codebook, which SH then applied to all transcripts. PA audited 20% of transcripts (*n* = 4) to check for consistent application of codes. Areas of differing coding were discussed and resolved using consensus-based discussions. PA and SH continued meeting to discuss transcripts and to analyze and generate themes together.

After applying codes, we used the Ottawa Decision Support Framework (ODSF) to frame the codebook and emerging themes. ODSF was created at the University of Ottawa in the 1990s to support practitioners and patients with difficult medical decisions. The framework emphasizes: (1) Identifying decisional needs, (2) Providing decision support based upon these needs, and (3) Reflecting upon decision outcomes. The ODSF has been utilized for a broad range of complex medical decisions including management of cancers, surgical decision-making, prenatal screening, and more (47). For its 20th anniversary, the authors performed a meta-analysis of randomized controlled trials that utilized the framework to create patient decision support aids, which found decreased decisional conflict and increased congruence between values and decisions in the ODSF arms (48). Themes were classified into three key domains guided by ODSF: the decisional impact of undergoing hysterectomy, the status of decisional support, and outstanding decisional needs.

## Results

### Participant demographics

A total of 20 women were interviewed, all of whom lived in and underwent hysterectomy in the Mysore District. The median age at time of interview was 38 years old, and the median age at hysterectomy was 25.5 years. All participants were Hindu and currently or previously married, although two (10%) were widowed and one (5%) was divorced. All had children, with a median of 2 children. The majority of participants had completed some schooling (85%), but none had completed post-secondary education. Most participants were homemakers (65%). Two participants (10%) were community health workers, Accredited Social Health Activists (ASHAs). Among participants' husbands, most worked as laborers in agriculture (*n* = 8, 40%) or as daily wage laborers (*n* = 7, 35%). In terms of education, most husbands of participants were illiterate (*n* = 5, 25%) or had attended only primary or high school (*n* = 12, 60%). Participants reported a range of annual household incomes, with a median of 100,000 rupees (approximately 1,200 USD). All participants reported undergoing hysterectomies at private nursing homes, with the exception of one participant who could not recall the location of her surgery. Demographic data are summarized in Table 1.

**Table 1:** Demographics of participants interviewed regarding experiences with hysterectomy at a young age in Mysore, India.

Demographic characteristic	Number of participants (*n* = 20)
Age in years, median (range)	38 (27–47)
Age in years at hysterectomy, median (range)	25.5 (19–35)
Years since hysterectomy, median (range)	13 (1–19)
Annual household income, median (range) *n* *=* *19, 1 unknown*	100,000 (10,000–350,000) Indian rupees
Number of children, median (range)	2 (1–3)
Marital status	
Married	17 (85%)
Widowed	2 (10%)
Divorced	1 (5%)
Education level	
Illiterate	3 (15%)
Primary school (standard 1–4)	3 (15%)
High school (standard 5–9)	9 (45%)
Secondary school (standard 10–12)	5 (25%)
Employment	
Homemaker	13 (65%)
Part time agricultural work	2 (10%)
ASHA^a^	2 (10%)
Other full-time work	3 (15%)
Husband's education level^b^	
Illiterate	5 (25%)
Primary school (standard 1–4)	2 (10%)
High school (standard 5–9)	10 (50%)
Secondary school (standard 10–12)	3 (15%)
Husband's employment^b^	
Agriculture	8 (40%)
Laborer	7 (35%)
Business	2 (10%)
Other	3 (15%)
Location of hysterectomy	
Private nursing home	19 (95%)
Unknown	1 (5%)

a Accredited Social Health Activist.

b Includes husbands of women who were divorced or widowed at the time of interview, given they were married at the time of hysterectomy.

### Themes

Our analysis revealed eight themes under three domains of participants' decision-making experience. Exemplar quotes are presented following each domain.

#### Domain 1: What is the decisional impact of undergoing hysterectomy?

*Theme 1. Alignment of personal values with decision-making leads to positive outcomes*

Many participants felt that undergoing hysterectomy was positive for them. For many, chronic illness and suffering were alleviated by the surgery (Quote 1). Hysterectomy was seen as an effective definitive management of chronic problems (Quote 2), which was especially important for women who had been concerned regarding possible life-threatening illnesses. Individual values varied regarding the importance of uterine preservation, but when other values aligned this was not always seen as crucial (Quote 3). Many women specifically discussed the freedom associated with avoiding restrictions on menstruating women in the local culture, which limited their entry into temples and kitchens during menstruation (Quote 4). The ability to work freely without suffering was important for many participants (Quote 5).

*Theme 2. Misalignment of personal values and decision-making leads to regret*

In contrast, other participants regretted hysterectomy when the outcomes did not align with their personal values. Regret surrounding loss of fertility was a common sentiment, with some women sharing they would have had more children if they had not undergone hysterectomy (Quote 6). Many felt they did not understand the expected physical changes in their bodies, which created more suffering than they were experiencing before the surgery (Quote 7). In contrast with those who felt freer to work after the surgery, many participants felt their health changes after hysterectomy now limited their ability to work (Quote 8). Some stated they would have made a different choice, had they had a better understanding of the outcomes of hysterectomy (Quote 9).

*Theme 3. Cost of medical care is a significant consideration for women with limited resources*

For most of the participants, cost was an important component of their experience with hysterectomy. Both before and after the surgery, chronic symptoms caused women to utilize the healthcare system, with time and money spent on appointments and treatments. For some, stopping these costs was a strong reason to pursue hysterectomy (Quote 10). On the other hand, the cost of the surgery itself was large, and many participants required help from family or others for assistance with paying (Quote 11). Women discussed weighing the cost of ongoing utilization of healthcare vs. a large one-time cost with hysterectomy (Quote 12).

Exemplar quotes for Domain 1 are presented in Table 2.

**Table 2:** Exemplar quotes for decisional impact among young women who underwent hysterectomy in Mysore, India.

Quote number	Participant ID	Quotation
*Theme 1. Alignment of personal values with decision-making leads to positive outcomes*		
1	8	Participant: I had so many problems at home, and I was not able to celebrate. It was a good decision that I did my surgery.
		Interviewer: If you had changed your decision that you are not going for surgery, what alternative you would have thought of?
		Participant: I would have spent my life eating medicines.
2	15	Even after the operation I worked well. If the operation was not done, I would have again went to the hospital, again they would have insisted to get operated, after that again I should have taken 6 months rest, so it's better that everything has been over now.
3	6	I don't have pain or anything so I'm happy. Other than the feel that I don't have my uterus, I don't have any other emotional feelings about it.
4	11	Previously I used to suffer each and every month. Now nothing like that. I'm ok. At the moment, I'm happy and peaceful. No periods. I can freely go and come anywhere or roam everywhere. I don't have to worry while going for temple as I won't get periods. I don't have to worry to go for a marriage or any other function. I just have to get ready and go, that's it.
5	16	After the operation, my health is better, it helped me in one or the other way. I was not able to attend any functions during my period time, I was not allowed to temple, even for kitchen. Now I don’t have such problems. My husband is ok, I am ok with it, before I could not work for more hours because of the pain. Now it's not like that, I am doing all the household work without any problems.
*Theme 2. Misalignment of personal values and decision-making leads to regret*		
6	12	If I had a uterus or if I had protected it, I could have one more child and it would bring me happiness. If I did not have any problem, then I would have had another child. I get tempted to have one more child when I see other children. That is not possible because I have got the uterus removed. I regret my decision of getting the operation done now.
7	2	If this operation was not performed, I would have led a better life. All these kinds of pain would not have occurred. For one pain I went and underwent the operation, now I am facing 16 types of pains.
8	18	I feel tired for every half an hour, or if I work a lot, I can't stand for more hours. I will get back and leg pain. I feel I should have said no to the operation. If I went to any other hospital they would have treated me with medicines, or maybe any other solution other than operation.
9	14	I knew nothing. If I had information, I would have somehow controlled my stomachache and would have not opted for hysterectomy.
*Theme 3. Cost of medical care is a significant consideration for women with limited resources*		
10	6	Due to this problem itself I have spent lakhs of rupees. I used to only eat tablets. No peace of mind and severe pain. Now also I had too much pain and started becoming too weak. It was necessary to get operated, so I underwent with the operation.
11	4	My husband was going for construction work and was also ploughing the lands for money. He did not have money. He mortgaged my jewels and bought the money for my operation. He had also taken loans from few people.
12	11	Bleeding used to increase every month. Every month we used to go to doctor and spend 1,000 or 2,000. They used to give tablets and two or three days it used to be ok. Again, next month same routine. Every time we used to spend 2–3 thousand. So we decided to spend at a time 30,000 and to be free with all these things and to get operated.

#### Domain 2: What is the status of decisional support, and what are the gaps?

*Theme 4. Women reflect upon their decision-making as inexperienced and rushed despite the long-term effects of a hysterectomy*

Regardless of whether they felt their decision was correct, many participants described their decision-making process as underinformed and often rushed. Women commonly reported being told they had a dangerous problem which needed to be addressed within days or their life could be at risk. Decisions in this context were often driven by fear and urgency (Quote 13). Gynecologic symptoms were seen to have the possibility of leading to cancer, and fear of cancer motivated them to pursue definitive treatment (Quote 14). Outside of personal fear, participants reported fear over the wellbeing of their families if they had a life-threatening illness (Quote 15). Even for participants who made the decision over a longer period, it was often at least in part affected by fear.

Many participants reported the time pressure contributed to making a less-informed decision, without sufficient information or a second opinion (Quote 16). Some described themselves as young and inexperienced with making significant medical decisions. Many women shared they did not fully understand the surgery they were getting or what was being removed from their bodies (Quote 17). One noted she was not aware that a hysterectomy would stop menstruation (Quote 18). Several participants reported that their illness was caused by their appendix, but the uterus was also removed (Quote 19).

*Theme 5. The decision to undergo hysterectomy is made with support from both medical and community resources*

The families of participants played a crucial supportive role for many of them in their illness and decision-making. Many were encouraged by family members to seek medical care for their symptoms (Quote 20). Most participants specifically highlighted the role of their husband in supporting them during their illness, helping them decide whether to pursue surgery and arrange for payment. Outside of the family, many participants also received information and advice from neighbors and other women in their communities who had undergone hysterectomy (Quote 21).

Despite their dissatisfaction with the surgery, participants felt the ultimate source of information regarding hysterectomy should be doctors. Participants noted respecting the doctors' opinions—one even calling doctors godlike (Quote 22). Many reported that doctors were the only trusted source of health information, especially a local doctor who knew them and their family. Participants deferred to doctors' authority in recommending the surgery (Quote 23).

*Theme 6. Participants recommend more formal support in place for other women who are considering hysterectomy*

Given their own experiences, many women reported the need for better decisional support for hysterectomy surgeries. Women often discussed during the interviews that “someone*”* should have better explained the surgery (Quote 24). Some highlighted that other women in their communities could also serve as potential sources of information and could often be unbiased compared to doctors (Quote 25). Many also emphasized the potential role of community health workers (CHWs) to fill this gap. In the Indian public health system, CHWs already provide information and support surrounding other aspects of reproductive health, and some suggested adding hysterectomy as a topic for them to focus on (Quote 26).

For many participants, however, the duty to counsel and educate patients remained with doctors. Some highlighted their own discomfort with giving women in their communities advice despite having gone through the surgery themselves. One participant, herself a CHW, noted that she was not qualified to give medical advice and recommended for women to seek care from doctors (Quote 27). Many also recommended that women take their time to decide, ideally with advice and opinions from multiple doctors (Quote 28). Overall, participants felt that doctors needed to take the responsibility to provide more thorough and balanced information regarding the risks and alternatives for hysterectomy.

Exemplar quotes for Domain 2 are presented in Table 3.

**Table 3:** Exemplar quotes for decisional support among young women who underwent hysterectomy in Mysore, India.

Quote number	Participant ID	Quotation
*Theme 4. Women reflect upon their decision-making as inexperienced and rushed despite the long-term effects of a hysterectomy*		
13	17	We had problem. Since they had given one week time and if in case we had not turned up there was threat to life, we took a loan and got it done.
14	20	Doctor had told my mother that the life of your daughter is at risk. Then my mother decided life is important more than that. I had 3 children. Excess of bleeding and white discharge might turn into cancer.
15	9	I had no other option, and I took medication also, but it did not help. If I die who will look after my kids and my husband? If my husband marries again who will take care of my kids?
16	18	If they would have said take medicine or consult any other doctor, I would have done that, but they didn't say anything regarding that. They said immediately the operation should be done, even I didn't have any one as of now to guide me.
17	2	Interviewer: Now when you are getting a hysterectomy done have the doctors told you, “this is the uterus, there will be so many parts, and this part will be removed”?
		Participant: They have not told me anything like that madam. When I went to them, while not feeling well they did the usual checkup and told they will admit me and do the operation, but they did not tell anything like this. We did not know anything like this.
18	18	After the operation for a few months, I used to think, “why am I not getting my periods?”
19	16	I had appendix, I had lot of bleeding and stomachache, so they said if it is not treated it may affect to uterus, so they did the operation (..) They said because of the appendix it was infecting all the parts, so after they said that, within three days I went through the operation.
*Theme 5. The decision to undergo hysterectomy is made with support from both medical and community resources*		
20	1	At that time my husband and mother-in-law said that I'm becoming very weak, can't see, and said, “let's consult a doctor.” Even my dad said that he's not able to see me in such condition and to go to a doctor.
21	19	My neighbors have undergone this operation. They have told me. (…) They told me that if we are getting stomach pain during periods, then it is better to get the operation done.
22	3	Nothing bad will happen to the doctors because they are equal to God. They save our life madam. Can we treat ourselves at that time, they treat us. We think they are equal to God and believe that they have lucky hands.
23	20	Madam, think, you're a doctor, I come to you saying I have stomach pain, if you give me medication or injection I can take it. If you tell me that I have to go through an operation, then I need to take up that decision to get it done.
*Theme 6. Participants recommend more formal support in place for other women who are considering hysterectomy*		
24	20	I didn't have anyone who came like this and explained it to me. There was no one to give me suggestions. I don't know what doctor's intention was. As the doctor stressed us, so there was no other go, we had to go through this operation. (..) I would have changed my decision if someone would have guided me in a right way.
25	7	If the woman asks the doctor, he will say the operation has to be done. So, she has to ask some other women.
26	13	Who should have given the information to us madam? The government gives information to post-delivery woman through *Anganwadi* workers. The same should be even done for the hysterectomy woman. They should also tell us that woman should not get this operation done.
27	17	I am doing social work in the village. In general people will be telling me, “I am not getting my regular periods, and it is for once in 2 or 3 months, there is excess bleeding, excess white discharge,” and so on. We cannot give treatment but say a few words. We cannot give tablets and medicines we can only suggest them to go to the doctor, take their opinion, never ignore, it might turn to some other disease in future.
28	12	When we go to a doctor, and if he says we have to get the operation done immediately, we should not get it done but should get it checked up in another hospital. We should wait and take the decision properly. We should compare both the things and then take a step forward. We should not just consider the opinion from one hospital but tally it with others also. We should think first and then decide.

#### Domain 3: What are the decisional needs that need to be addressed for individual women?

*Theme 7. Knowledge and expectations of the outcomes of surgery need to be personalized and accurate*

In their call for improving decisional support, participants highlighted information as a key decisional need. Many felt their knowledge was limited, and therefore they may have had inaccurate expectations for the outcomes of hysterectomy surgeries (Quote 29). Notably, participants often cited heavy vaginal bleeding, chronic abdominal pain, and abnormal vaginal discharge as chronic health issues that led them to hysterectomy. Women shared that their decision to undergo hysterectomy would have been changed with more information regarding their illnesses and which alternative treatments may have been available (Quote 30). Many participants emphasized that hysterectomy was not a one-size-fits-all solution as they may have believed before undergoing the surgery themselves, but rather the decision to undergo surgery must be individualized. They highlighted that each woman's medical scenario may be different and require different treatment (Quote 31). Many noted that women undergo hysterectomy for non-life-threatening illnesses without appropriate expectations of what else may change in their life after the surgery. Participants encouraged women to understand the outcomes of hysterectomy and only undergo the surgery if the benefits outweighed possible poor outcomes (Quote 32).

*Theme 8. Personal values regarding uterine preservation, fertility, and ability to work should be clarified for women to make appropriate decisions*

The varying values of participants emphasize the importance of exploring these values to make individualized decisions. Participants discussed the common idea that the uterus is an integral source of strength and health for a woman. Many felt that their physical strength had decreased since their hysterectomy (Quote 33). For some, losing this organ affected their view of their womanhood (Quote 34). Many women felt that the importance of the uterus is such that they attributed health problems which developed even years later to their hysterectomy (Quote 35). Participants discussed the idea that menstruation is a necessary process for removing toxins or other negative components of the body, and that lack of menstruation can contribute to bad health and weight gain (Quote 36). For others, limitations placed on them during menstruation made lack of menses a welcome change (Quote 37).

Similarly, women varied in their reaction to infertility due to hysterectomy. Many had completed their families prior to the surgery, so that this was not a consideration for them. Some participants delayed their hysterectomy until their families were complete (Quote 38). However, for those who lost fertility, it could have devastating effects. One participant subsequently got divorced and felt her ability to remarry was negated due to the surgery (Quote 39).

Finally, for many women, discussions of health and wellness were intertwined with their ability to work, take care of their children, and otherwise contribute to their households. For some, the desire to work was a driver for pursuing hysterectomy in the first place, as a definitive way to manage their chronic health conditions. When ability to work was improved, women felt positively about undergoing hysterectomy (Quote 40). Other participants felt weaker after the surgery and less able to contribute to work (Quote 41). The changes in strength and energy were commonly described as laziness (Quote 42).

Exemplar quotes for Domain 3 are presented in Table 4.

**Table 4:** Exemplar quotes for decisional needs among young women who underwent hysterectomy in Mysore, India.

Quote number	Participant ID	Quotation
*Theme 7. Knowledge and expectations of the outcomes of surgery need to be personalized and accurate*		
29	4	I had seen other women who had undertaken this operation, and even I had white discharge. Some of them told me that if it is removed quickly then the pain will be less, but they did not tell me that there will be suffering even after the operation. It will be fine in the beginning but later it gets turned to a lot of pain. It should be done in the right age.
30	10	We don't have too much knowledge. In my case, if I would have had knowledge, I would have asked them to remove only appendix and not uterus. In my family they were afraid thinking if not operated she might expire. They thought if it is removed, she will be well and good and healthy.
31	19	Participant: One woman came to me and asked me why I got the operation done. I told her that I got the operation done because I had discharge and lower-back pain. She told me she also had the same problem.
		Interviewer: Did you tell her to get the operation done?
		(..) Participant: Everything is dependent on their body. My body is different from hers. We cannot be the same.
32	2	Due to this we cannot do any other work, our health will not be good. (…) If the problem cannot be cured unless they get it operated, let them go for it. They should not get the operation done for minor petty reasons.
*Theme 8. Personal values regarding uterine preservation, fertility, and ability to work should be clarified for women to make appropriate decisions*		
33	4	Once a cow had pulled me and I had no strength to pull it back with force. I have become weak. I have become like a tree with its roots cut. Uterus for women is like the roots for a tree and is very important.
34	17	I have lost an organ from which I was recognized as a woman. I feel bad. I should have retained my uterus is what I feel. Uterus is very important for a woman. You can live without arms or legs. Uterus is the gift given by God to a woman which I lost. I feel very bad.
35	1	After the operation until one year I didn't have any problem, I felt, “I'm ok.” After coming here and working I started getting knee pain, back, pain, and many other problems one after the other. I felt at that time, “why did I go through this operation?”
36	14	I have no other problem, but when a woman gets her period all the bad things in her body will come out, but when she is not getting her period all the bad things get collected in the body creating so many problems.
37	16	During those 5 days we should not go to kitchen, at that time it was quite difficult for our husband and children. No one was there to cook, now I don't have such tension I can go and take care of all those things.
38	9	I thought only one daughter is enough, and if I had more kids, by any chance if I die who will look after my kids. My cousins said they will look after my kids, so I gave birth to another baby boy. When he was 6 months old doctor told me to get operation done and I got it done.
39	17	I feel I should have had my uterus. I feel sad. (…) If I had my uterus my life would have been different. (…) After we separated, I could have married. I lost that, I spoilt my life myself. I separated at the age of 20 years. If I had my uterus I would have got a life, I lost my life.
40	9	Before operation my husband used to look after my kids like bathing them. After the operation, I am able to do all the work now. I have to thank that doctor million times. I now even go to field and work.
41	18	My health was in good, I worked for more than an hour, but now even if I work for half an hour I feel tired. I cannot eat properly, I will get leg pain, back pain. I feel that, “why did I get through the operation?”
42	13	I was earning nicely earlier. I was not getting tired or pain. Now I feel my body is very heavy. I do not feel like working. I feel lazy. I do not feel like having food. For all these reasons I feel I am unhealthy.

## Discussion

Our study findings highlight the complexity of decision-making for a major surgery with implications for physical health, fertility, and social wellbeing among Indian women. The diversity of their attitudes towards hysterectomy based on personal experiences and values emphasizes the need for this decision to be made on an individual basis with appropriate personalized support.

**Figure 1:**
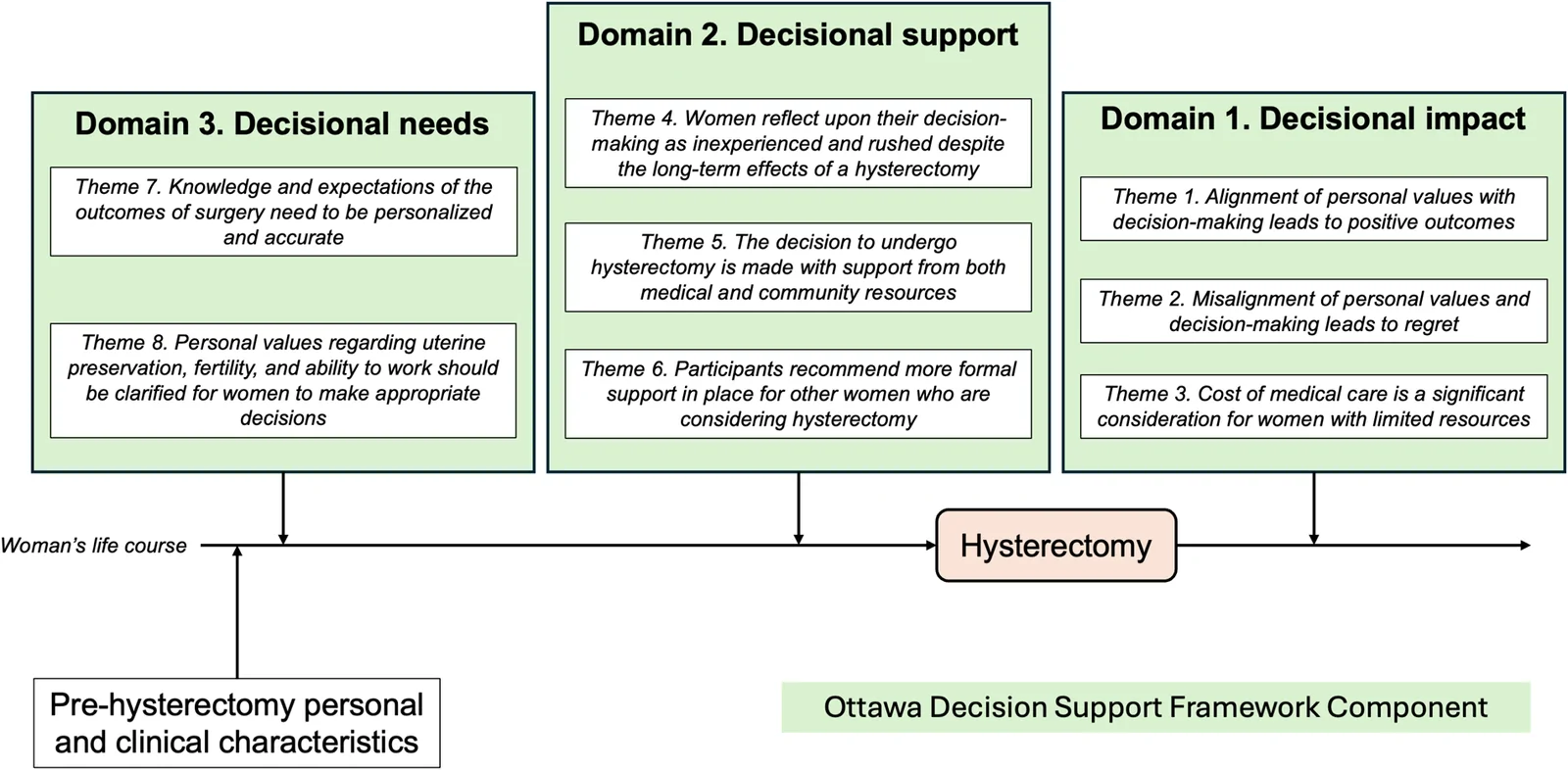
Hysterectomy decision needs framework for young women in Mysore, India.

**Figure 2:**
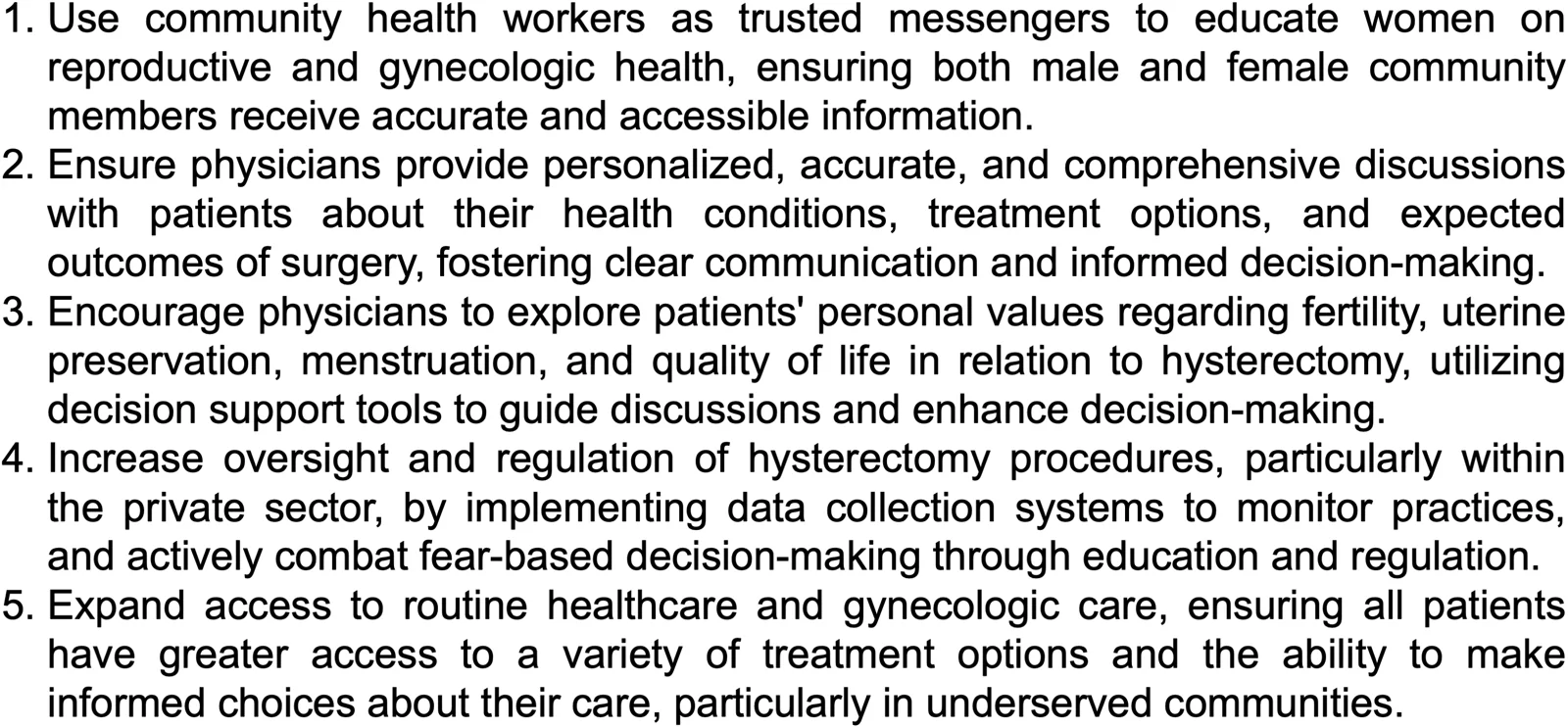
Recommendations for improved hysterectomy decision support from young women in Mysore, India.

Applying the ODSF to the participants' experiences helped identify the gaps in decision-making needs (Figure 1). Undergoing hysterectomy for benign conditions is the correct choice for some women, but our findings highlight the current lack of decision support, and a large proportion of participants experienced decisional regret. Our participants attributed this regret to the gap that currently exists in decisional support and provided important recommendations for how to improve this. We have summarized these recommendations in Figure 2.

First, women described a strong need for information and education regarding hysterectomy and gynecologic health in general, from both CHWs and physicians. CHWs' role was described as providing education on reproductive health and hysterectomy in general. Given the support women received from neighbors and family members as well as the strong societal norms surrounding hysterectomy, it would be important for not only individual- but also community-level education. Specifically, participants discussed male family members like their husbands, fathers, and others engaging in decision-making support and consideration of finances. In deeply patriarchal settings, women's reproductive choices are not only medical but also socio-cultural. Interventions must therefore address broader gender norms through male-engagement programs, community dialogues, and gender-sensitivity training for providers to shift the underlying power dynamics that drive both demand for and delivery of hysterectomy. Community-level interventions should include education regarding gynecologic health addressed towards women and men. Such education interventions delivered by CHWs are supported across the spectrum of preventive and curative health services in India (49).

Physicians were viewed as the essential source of personalized health recommendations and information to address decisional needs, and a very trusted source of information. Participants highlighted the important role of physicians to provide specific, accurate, and thorough information to individual women regarding their health problems, likely causes, and treatment options to come to the best decision for them. Furthermore, participants reported inconsistent discussions with physicians regarding personal characteristics and values such as fertility and/or family completion, preservation of the uterus, and desire for menstruation. Participants often framed discussion of health and wellness around their ability to care for their families and work, however it was unclear how much these values were discussed with providers. Overall quality of life was extremely important to participants, and they attributed much of their current quality of life or lack thereof to their hysterectomy. However, they consistently reported this was not discussed with them prior to surgery. Physicians need to explore these values with women considering hysterectomy to appropriately counsel them. Although we are not aware of any currently available in *Kannada*, physicians may consider using decision-support tools to provide patients with a framework for decision-making (50, 51).

The salient theme of urgent fear-based decision-making emphasized how gaps in decision support directly lead to decision regret. Furthermore, all participants in the study who could recall the location underwent hysterectomy at private facilities, where costs were often significant, and which may have different financial incentives than public facilities. Many participants reported requiring loans or support from additional family members due to their limited resources and the stated urgency of the surgery. The combination of fear-driven decisions to undergo a high cost procedure aligns with previously reported concerns regarding potentially coercive approaches to pre-hysterectomy counseling (40). Future policy work should examine how fee-for-service models and insurance reimbursements incentivize high-volume surgical care and explore alternative payment reforms such as bundled payments or performance-based incentives that reward quality counseling and conservative management, where appropriate. Participants' experiences underscore the need for government-level expansion of oversight from regulatory agencies to monitor and react to overuse of hysterectomy in public and private healthcare facilities.

Lastly, participants' utilization of hysterectomy for medical problems including chronic abdominal pain, appendiceal illnesses, heavy periods, and vaginal discharge demonstrates the current gap in access to appropriate healthcare in general. While we relied upon participants' recounting of their own experiences and therefore have limited insight into their medical conditions, there is no doubt that some of these pathologies (appendicitis, vaginitis) are not addressed by hysterectomy. For those that may be, for example heavy bleeding and pain due to uterine fibroids, it is likely that access to ongoing care to address these problems may have avoided some hysterectomies. The theme of seeking a one-time cost with hysterectomy over recurring expenses of care for chronic conditions highlights that appropriate care must also be affordable. Furthermore, integrating care for mood dysfunction, chronic pain, and complications of weight gain may be necessary to address the difficulties participants reported in their quality of life both before and after hysterectomy.

Our findings align with previous work around the world regarding hysterectomy, decision-making, and regret. Data from the United States, Scotland, and Canada have repeatedly shown that women want more information and discussion of all options to participate in shared decision-making with providers (52–56). Increased knowledge pre-operatively correlates with women feeling hysterectomy was concordant with their values (57), and using shared decision-making tools has high levels of satisfaction and concordance with values (50, 51). Perhaps most notably for our patient population, a survey of women who underwent hysterectomy under age 35 in Canada found low levels of regret when women felt autonomous in decision-making, with appropriate counseling and previous trial of alternative medical therapies (58). Piloting low-literacy, pictorial decision aids delivered by CHWs, adapted from the Ottawa tools and tested in *Kannada* could be a scalable way to embed shared decision-making into routine community outreach. Implementation research should assess acceptability, feasibility, and impact on decisional conflict.

The fear-based decision-making reported by our participants aligns with that found by Xavier et al. (40). During focus groups with women in the Chikkamagaluru and Gulbarga districts of Karnataka, participants shared that doctors often instilled fear of cancer, told them they needed urgent surgery within days to prevent bad outcomes, and followed up post-operatively for more referrals. They also similarly reported women undergoing both appendectomy and hysterectomy for abdominal pain.

As highlighted by Desai et al. with their extensive work on hysterectomy in Gujarat, women in India often seek hysterectomy as definitive management because they live in a healthcare system with limited access to ongoing medical care (17). This has been supported by work in Maharashtra (22), and has been suggested to drive high hysterectomy rates among rural women in Tamil Nadu (59). Our findings in a sample of women from rural and semi-urban areas in South India concur with this prior work. Desai's group have found that while education has been shown to increase knowledge among women regarding hysterectomy, in isolation it does not change behaviors (25). Our recommendations align with theirs, which emphasized that while education and counseling for individual women are important, fundamental structural changes must be made in the delivery of healthcare for Indian women (23). Amid calls for change such as these, the Indian Ministry of Health and Family Welfare has recently published a Guidelines to Prevent Unnecessary Hysterectomy which details programmatic guidance for local public health officials and clinicians, including recommendations for improving public awareness, requirements for monitoring and data collection, and clinical protocols (60). While an important first step, rollout of these efforts must be consistent and effective to change current public and clinician attitudes toward hysterectomy. As these national guidelines are operationalized, it will be critical to define clear indicators for rates of pre-surgical counseling, uptake of conservative therapies, and regional hysterectomy trends to monitor both intended improvements and unintended consequences (e.g., under-treatment of serious pathology). Furthermore, as oophorectomy has serious implications for long-term post-operative health outcomes in addition to those associated with hysterectomy, specific monitoring and oversight of concurrent oophorectomy are critical to incorporate. To translate recommendations into practice, future qualitative work should map decision pathways from multiple vantage points including gynecologists, primary care physicians, insurance officials, and public-health leaders to identify system-level enablers and barriers to more equitable, patient-centered care.

These findings are based in the strengths of our study. To our knowledge, this is one of the few studies using a theoretical framework in health care decision-making to explore and better understand Indian women's decisional needs when making decisions around hysterectomy. Second, by employing a qualitative approach, our study findings highlight the complexity of the decision-making process around hysterectomy, specifically in the context of Indian women. Finally, by including the perspectives of the women who have undergone the surgery, the study provides findings and recommendations are grounded in the voices of individuals most affected by the hysterectomy surgery.

We acknowledge limitations, including that our study is intentionally focused on women in the semi-rural areas surrounding Mysore who were all Hindu. While we believe the literature suggests their experiences are relevant for other women in India, we acknowledge that nuances of their experiences are likely not universally applicable. Furthermore, we used snowball sampling, which is vulnerable to sampling bias including first wave bias, in which the characteristics of the initial participants can disproportionately influence the entire sample, potentially excluding other important groups. There was over 10 years difference between the median ages at time of interview and at hysterectomy, which highlights the potential for recall bias regarding decision-making. While we aim to capture women's experiences with hysterectomy, we are therefore limited to this viewpoint and do not have additional medical information such as their specific diagnoses or which alternatives would have been available. This limits our ability to assess the appropriateness of hysterectomy for participants. Additionally, given our focus on women's own perspectives on their experiences, we did not collect any validated measures of quality of life outcomes including mental wellbeing, which may be of utility in future research on hysterectomy decision making and outcomes. We did not interview women who considered hysterectomy but did not undergo the surgery, which would be another important perspective to understand decision-making. Additionally, these recommendations are made based on patient experiences, but further work exploring additional stakeholder perspectives (physicians, community health workers, public health officials) is necessary to better understand their roles and limitations.

In conclusion, the study findings reveal significant gaps in the decisional needs among women considering hysterectomy. For many women, the fear of health conditions like cancer drove decisions that were made in haste and often lacked critical information regarding the operation, its risks, and alternatives. Analyses conducted in this study guide the recommendations for future policy, practice, and research efforts. These recommendations include CHW- and physician-driven knowledge and education efforts, in-depth exploration of personal values among women, and policy approaches to improve regulation of hysterectomy in the private sector and to expand access to healthcare for gynecologic and non-gynecologic conditions to prevent unnecessary hysterectomies. Finally, to ensure these recommendations translate into real-world impact, future efforts should establish ongoing monitoring and evaluation frameworks, co-designed with women, providers, CHWs, and policymakers that track both patient-reported decision quality and system-level hysterectomy trends over time.

## Data Availability

The raw data supporting the conclusions of this article will be made available by the authors, without undue reservation.
