# Supplementary material for: “Someone could have told me”: a qualitative study on experiences with hysterectomy decision-making and regret in Mysore, India
Source: Front Glob Womens Health. 2026 Aug 10;7:1870806. doi: 10.3389/fgwh.2026.1870806 (PMC13500569; doi:10.3389/fgwh.2026.1870806)
Supplement: Supplementary file 1 [file Supplementaryfile1.pdf]

SH is from the United States and identifies as White. She lived in Mysore during the study period but is not from this area and does not speak *Kannada*. She was a medical student at the time of the study and is now an Infectious Diseases physician-scientist. She acknowledges her involvement in the study and interpretation of the data are affected by her perspective as an outsider to the studied communities and her position of privilege. She comes to this work from a place of valuing health justice and advocacy for women's health in resource-constrained settings.

RK has over 25 years of clinical experience in India in various capacities and roles, currently serving as Research Physician and Gynecologist at Public Health Research Institute of India, Mysore. She is an Indian citizen. She follows the Hindu religion and speaks five Indian languages: Kannada, English, Hindi Tamil and Telugu.

RP, FB, and PJ self-identify as Asian/Indian women who live and work in Mysore, India. They have ongoing professional relationships working long-term in the communities which were sampled. They speak English and Kannada.

VS is an Indian physician specialized in Obstetrics and Gynecology. She served as Research Physician at the Public Health Research Institute of India for 15 years, providing services for the communities studied in this project. She speaks English and Kannada.

RH is a public health researcher in a government organization, trained in qualitative methods and health systems research. As an Indian woman who speaks English and Hindi, she is particularly concerned about unnecessary hysterectomies without clear clinical indication, which shaped her interpretation of the qualitative data.

PM is a physician-scientist with a background in women's health in limited resource settings. This project, focusing on understanding the decision-making process for women undergoing hysterectomy in India, is a result of an over of decade of her work in women's health. She now resides in the United States and acknowledges that she is a researcher operating from a position of privilege and may have shaped her perspective of this work that was conducted. She recognizes the power dynamics inherent in this relationship, where her role as a researcher could potentially influence the interpretation of the findings.

PA brings her training and perspective as an implementation scientist with expertise in cancer prevention. Her work spans both local and global contexts, often in resource-limited settings, and focuses on ensuring that evidence-based interventions in cancer prevention are implemented equitably so that all populations benefit. She uses quantitative, qualitative, and mixed methods. She is an immigrant to the United States from India and a resident of Albuquerque, New Mexico, living and working on the traditional lands of the pueblos and tribal nations. This positionality also deepens her own commitment to reciprocity and community engagement in research.

DS is an Obstetrician/ Gynecologist with over 25 years of clinical experience in the U.S, Zambia, Guatemala, and India. She is of Indian origin, holds U.S. citizenship, and has been living in India since 2022. She follows the Hindu religion and speaks four Indian languages: Hindi, Gujarati, Kutchi, and Kannada.
