# Supplementary material for: “Someone could have told me”: a qualitative study on experiences with hysterectomy decision-making and regret in Mysore, India
Source: Front Glob Womens Health. 2026 Aug 10;7:1870806. doi: 10.3389/fgwh.2026.1870806 (PMC13500569; doi:10.3389/fgwh.2026.1870806)
Supplement: Supplementary file 2 [file Supplementaryfile2.pdf]

Interview Discussion Guide

The overall goal of the study is to explore why women in Mysore District receive hysterectomy and the effects of the surgery on their lives.

Human rights framework: decision-making in the light of autonomy, dignity, informed consent, equality, and access to care.

STAGE 1: Introduction/ Icebreaker

1. Can you tell me about your family?
   - Who lives here at home with you?

Icebreaker, gives context for later questions about lifestyle and relationships.

STAGE 2: Decision to undergo hysterectomy

2. As you know, you are doing this interview because you have had a hysterectomy, can you tell me what that means to you?
   - *If needed, explain that it is a surgery to remove uterus, cervix, and sometimes ovaries and fallopian tubes.*
   - Can you tell me who needs this surgery? For what reasons?

Understand what she knows about hysterectomy, how she thinks about it.

3. So you had a hysterectomy, can you tell me about why you did it?
   - What made you decide to go ahead with surgery?
   - How did you learn about having a hysterectomy?
   - Who helped you to make this decision? Who did you discuss the decision with?
   - Did you have enough information to make the decision to get a hysterectomy?
   - Did cost affect your decision? How?

Understand her decision-making process: what was the medical reason, what information she needed to make a decision, how she got that information, whether she was primarily the one making that decision, who helped her, how did the surgeon vs. husband/family/ friends influence her?

4. Were there any other options you considered or tried doing before you had the hysterectomy?

- For example, did you try medical treatment, natural treatments, or Ayurvedic treatments?
- You had the hysterectomy because you were having (*her medical problem*), was there anyone else besides a surgeon who helped or could have helped you with that?
- How long did you try other options before deciding on hysterectomy?
- If you had not undergone hysterectomy what else would you have done?

Understand whether she knew about and/or tried alternatives before hysterectomy, who told her about those options, what her access to healthcare is like.

STAGE 3: After the hysterectomy

5. After the hysterectomy, can you tell me how you felt physically?

- How was your recovery time? Were there any difficulties?
- Who helped you during your recovery?
- Overall since the hysterectomy, has your physical health improved or gotten worse? In what way?

Understand her recovery, any complications she experienced, how prepared she and her family were for complications, whether her medical problem was resolved by the surgery, how she is feeling now, physically.

6. What about how you felt mentally, and your emotions after the hysterectomy?

- Have you felt happier or sadder or something else?
- Can you tell me about having anxiety or mood swings?
- How satisfied are you in your sexual life?

Understand how the surgery affected her mental health, whether she has been having menopause symptoms, changes in her sex life.

7. Now that you no longer get your period, how does that feel?

- Have there been any changes in your daily activities?
- Have there been any changes in your relationships?

Understand how menstruation and lack thereof affect her life, how her work and/ or family statuses have or have not changed.

8. How do you feel now that you won't be getting pregnant?
- Before the surgery, how confident were you that you did not want more children?
- Do you feel different knowing that you do not have a uterus?
- How does your husband or family feel about this?

Understand how fertility, having a womb made her feel, whether this was a factor in her decision making, how it has changed her relationships.

STAGE 4: Conclusion

9. Thinking back to your decision to get a hysterectomy, how do you feel about it now?
- If you could change the decision you made, would you? Why?
- If there were another woman in a similar situation to you, would you recommend that she should get a hysterectomy?

Understand what she thinks about her decision-making process, and her overall experience with having a hysterectomy, whether she wishes her experience were different and in what way.

10. What information do you think other women would need to know before having a hysterectomy?
- Would another woman need more information to prepare her for health changes or complications? If yes, what information and from where?
- What do you think that the surgeon should know to better serve other women in the future regarding hysterectomy?

Understand what kind of decision-making process she would have wanted, other information she was missing before the surgery, whether she understood the risks or complications, how she could be better served now post-op.

11. Is there anything else that you would like to add?

Any new insights we have not considered.
